# *De novo* transcriptome assembly and annotation for gene discovery in avocado, macadamia and mango

**DOI:** 10.1038/s41597-019-0350-9

**Published:** 2020-01-08

**Authors:** Tinashe G. Chabikwa, Francois F. Barbier, Milos Tanurdzic, Christine A. Beveridge

**Affiliations:** 1 School of Biological Sciences, The University of Queensland, St. Lucia, Brisbane, Queensland 4072 Australia; 2 Queensland Alliance for Agriculture and Food Innovation, The University of Queensland, St. Lucia, Brisbane, Queensland 4072 Australia

**Keywords:** RNA sequencing, Plant molecular biology, Transcriptomics

## Abstract

Avocado (*Persea americana* Mill.), macadamia (*Macadamia integrifolia* L.) and mango (*Mangifera indica* L.) are important subtropical tree species grown for their edible fruits and nuts. Despite their commercial and nutritional importance, the genomic information for these species is largely lacking. Here we report the generation of avocado, macadamia and mango transcriptome assemblies from pooled leaf, stem, bud, root, floral and fruit/nut tissue. Using normalized cDNA libraries, we generated comprehensive RNA-Seq datasets from which we assembled 63420, 78871 and 82198 unigenes of avocado, macadamia and mango, respectively using a combination of *de novo* transcriptome assembly and redundancy reduction. These unigenes were functionally annotated using Basic Local Alignment Search Tool (BLAST) to query the Universal Protein Resource Knowledgebase (UniProtKB). A workflow encompassing RNA extraction, library preparation, transcriptome assembly, redundancy reduction, assembly validation and annotation is provided. This study provides avocado, macadamia and mango transcriptome and annotation data, which is valuable for gene discovery and gene expression profiling experiments as well as ongoing and future genome annotation and marker development applications.

## Abstract (summary)

**Table:** 

Measurement(s)	transcription profiling assay • sequence_assembly • sequence feature annotation
Technology Type(s)	RNA sequencing • sequence assembly process • sequence annotation
Factor Type(s)	plant species
Sample Characteristic - Organism	Persea americana • Macadamia integrifolia • Mangifera indica
Sample Characteristic - Location	Australia

Machine-accessible metadata file describing the reported data: 10.6084/m9.figshare.11303135

## Background & Summary

Fruits and nuts are an important source of vitamins and dietary fibre for consumers and a source of income for farmers. Avocado (*Persea americana* Mill.), macadamia (*Macadamia integrifolia* L.) and mango (*Mangifera indica* L.) are important commercial tree species grown in Australia and other tropical/sub-tropical regions. In 2013, the world production of avocado was about 4.7 million tonnes^1^. Macadamia is grown commercially for its edible nuts in tropical and subtropical regions, including Australia, Hawaii, China, Thailand, southern and central Africa and Central and South America^2^. Mangoes are produced commercially in at least 87 countries on an estimated area 5 million hectares with an annual production of over 35 million tonnes^3^. Despite their commercial and nutritional importance, these tree crops are yet to benefit from a substantial research effort required to generate significant public bioinformatic resources. These resources are essential for functional genomics studies, marker-assisted breeding, cultivar development, and genome annotation efforts. Here, we report on the generation and availing of transcriptomic resources for avocado, macadamia and mango.

Currently a few genomic resources are available for avocado, mango and macadamia. Most of the publicly available *de novo* transcriptome assemblies of avocado and mango are limited to either leaf or fruit tissue^4–7^. Only two studies published open-access transcriptome assemblies from several tissues of avocado and mango respectively^8–10^. However, these assemblies were derived from RNA-Seq libraries that were not normalised and therefore lack some essential yet lowly expressed genes and near-universal single-copy genes (Supplementary Fig. 1). Additionally, the ‘Keitt’ mango transcriptome study^9^ was designed for SNP discovery and did not produce a reference transcriptome for gene discovery purposes. A reference macadamia genome assembly with its accompanying reference gene set was recently published^11^. However, this genome assembly comprises 79% of the estimated macadamia genome size^11,12^. A draft mango genome was published in 2016, although it is not yet be publicly available^13^. We believe that our *de novo* transcriptome assemblies derived from normalized RNA-Seq libraries are complimentary to these resources as they accentuate rare/low abundance transcripts. In eukaryotes, the high abundance transcripts (several thousand mRNA copies per cell) from as few as 5–10 genes account for 20% of the cellular mRNA^14^. The intermediate abundance (several hundred copies per cell) transcripts of 500–2000 genes constitute about 40–60% of the cellular mRNA. The remaining 20–40% of mRNA is represented by rare, low abundance (from one to several dozen mRNA copies per cell) transcripts^14^. Such an enormous difference in transcript abundance compromises gene discovery, which results in poor detection of genes transcribed at relatively low levels.

**Fig. 1:**
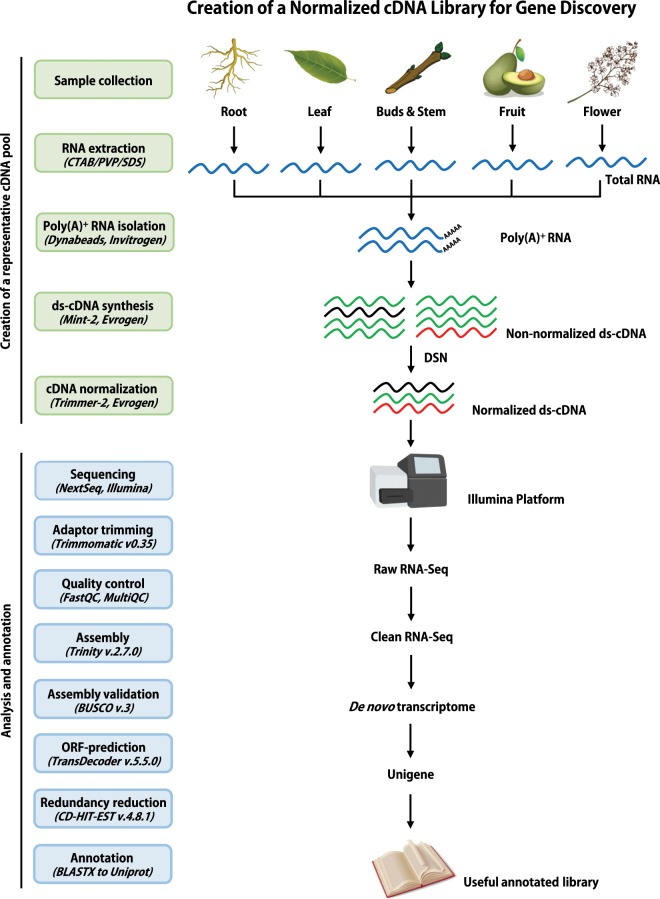
Flowchart of the CDNA library preparation, RNA-sequencing setup and *de novo* transcriptome data analysis steps (created with BioRender.com).

We therefore prepared comprehensive cDNA libraries from RNA pooled from of a wide range of plant tissues (leaf, stem, axillary bud, root and flower and fruit/nut) to maximize the number of transcripts represented in each library. The essential part of the library preparation process was converting the pooled RNA into normalized cDNA using a duplex-specific nuclease (DSN) normalization protocol^15^. This was done to avoid the dilution of transcripts from lowly expressed genes by those from highly expressed genes (Fig. 1) and therefore to improve gene discovery^16^. The assemblies generated in this study can be utilized as reference gene sets for a variety of tree genomics studies requiring transcriptome information of *Persea americana*, *Macadamia integrifolia*, *Mangifera indica* and related species. For example, considering that *Persea americana*, and *Mangifera indica*, are both prone to alternate/biennial bearing^17,18^, identification and subsequent manipulation of genes regulating floral induction may greatly contribute to solving this problem. Our transcriptome assemblies will also assist in mRNA-based genome annotation^19^ for ongoing whole genome sequencing projects of macadamia and mango^11,13^.

## Methods

### Sample collection

Tissue samples were collected from mature (7–15 year old) field-grown avocado cv. “Hass”, mango cv. 1243, and macadamia cv. 751 trees in Queensland, Australia. Plant tissue sampled included young and mature leaves, dormant and bursting axillary and terminal buds, mature and elongating stems and roots, a mixture of floral tissues at different stages of development and a mixture of fruit tissue in the case of avocado and mango or nuts in the case of macadamia. Fresh material was flash frozen in liquid nitrogen or dry ice and stored at −80 °C before being homogenized using an automated tissue grinder (Geno/Grinder®, SPEX).

### RNA extraction

RNA was extracted from the different samples using a CTAB/PVP/SDS method developed for these types of samples as previously described^20^. Briefly, frozen powder was lysed using a CTAB/PVP buffer + 1 mM DTT for 10–15 min. One percent SDS was then added to each sample before centrifugation for 15 min at 20,000 g. The liquid phase containing the nucleic acids was up taken and added to an equal volume of isopropanol before centrifugation (20,000 g) for 45–60 min at 4 degrees. The nucleic acid pellet was then washed with 70% ethanol and resuspended in water. DNase treatment was then applied for 25 min and RNA was precipitated in an equal volume of isopropanol to form a nucleic acid pellet. The pellets were washed in 70% ethanol and then resuspended in pure water. RNA concentration was measured using a NanoVue™ Plus Spectrophotometer (GE Healthcare Life Sciences, USA). RNA integrity check was performed by agarose gel electrophoresis.

### Normalised cDNA Library preparation

One normalised cDNA library was prepared for each of avocado, macadamia and mango, from equal amounts of mRNA from the different tissue types mentioned above and as described in Fig. 1. Poly(A)-RNA was isolated using oligo(dT) magnetic beads (Invitrogen™ Dynabeads™). 0.5–1 μg of the poly(A)RNA was converted into full-length-enriched double stranded cDNA using the Mint-2 cDNA synthesis kit and following the manufacturer’s instructions (Evrogen, Moscow, Russia). The double stranded cDNA was then normalized using the DSN-based Trimmer-2 cDNA normalization kit and following the manufacturer’s instructions (Evrogen, Moscow, Russia). The normalized cDNA libraries were then sheared into ~300 bp fragments with a sonicator (Bioruptor^®^, Diagenode) and indexed with adaptors using the NEBNext^®^ DNA Library Prep Master Mix Set for Illumina^®^. Four technical replicates of each of the three normalized cDNA libraries were sequenced on the Illumina NextSeq. 500 platform (Fig. 1) with the primary objective of enhancing *de novo* gene discovery.

### *De novo* assembly and dataset annotation

**Fig. 2:**
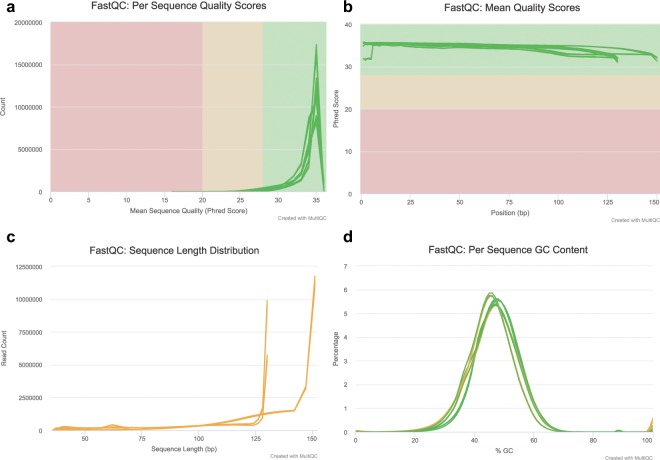
Quality assessment metrics for trimmed and filtered RNA-Seq data used to make the *de novo* transcriptome assembly.

High-quality RNA-Seq reads (sequences) were used in the subsequent *de novo* transcriptome assembly. Raw RNA-seq reads were pre-processed by removing adapters and low-quality sequences (<Q30) using Trimmomatic (v. 0.35) with default parameters^21^. Sequencing summary statistics showing the total number of reads before and after trimming and quality filtering is presented in Table 1. RNA-Seq read quality before and after trimming was assessed using FastQC (https://www.bioinformatics.babraham.ac.uk/projects/fastqc/) and aggregated using MultiQC^22^, read quality after trimming is presented in Fig. 2. *De novo* transcriptome assembly was done with Trinity (v. 2.7.0) using default settings^23,24^. Coding regions of the assembled transcripts were predicted using TransDecoder (v. 5.5.0) with default settings^24^. We used selected the single best open reading frame (ORF) per transcript longer than 100 peptides. We then used the CD-HIT-EST program (v. 4.8.1) with default parameters (similarity 95%) to reduce transcript redundancy and produce unique genes (“unigenes”)^25^. We used Basic Local Alignment Search Tool (BLAST) to assign functional annotations to the unigenes^26,27^.

**Table 1:** Read summary statistics and comparative analysis of Avocado and Macadamia RNA-Seq reads and *de novo* assembled transcripts to publicly available avocado and macadamia genomic resources.

	Avocado	Macadamia	Mango
NCBI BioSample accession numbers	SRR8926023, SRR8926022, SRR8926017, SRR8926016	SRR8926019, SRR8926018, SRR8926021, SRR8926020	SRR8926027, SRR8926026, SRR8926025, SRR8926024
Total number of raw reads	226341270	159438181	188997291
Total number of reads after trimming	209971284 (92.77%)	150743988 (94.57%)	167567866 (88.6%)
Reference genome size	912.6 Mbp	652 Mbp	N/A
Number of trimmed reads mapped to reference genome	166781058 (73.69%)	127314454 (79.85%)	N/A
Average depth of coverage of mapped reads	29.09	20.93	N/A
Reference gene sets (number of sequences)	24616	35337	N/A
Number of unigenes in *de novo* transcriptome assemblies	63420	78871	82198
Unique BLASTN matches to reference gene sets	22670 (92%)	27322 (77%)	N/A

Reference genomes and genesets used for the comparative analysis are Rendón-Anaya *et al*. (2019) Nock *et al*. (2016) for avocado and macadamia respectively.

## Data Records

Nine datasets were generated in this study. The first datasets consists of RNA-seq raw reads of *Persea americana*, *Macadamia integrifolia* and *Mangifera indica*, which were deposited in the NCBI Sequence Read Archive database under project identification number **PRJNA533518**^28^. Datasets containing *Persea americana*, *Macadamia integrifolia* and *Mangifera indica* transcriptome assemblies were deposited in the NCBI Transcriptome Shotgun Assembly (TSA) database under TSA accession numbers **GHOF0000000**^29^, **GHOE00000000**^30^ and **GHOG00000000**^31^. Datasets containing *Persea americana*, *Macadamia integrifolia* and *Mangifera indica* raw trinity transcriptome assemblies, unigenes, and functional annotation files were deposited in Figshare^32–34^.

## Technical Validation

**Fig. 3:**
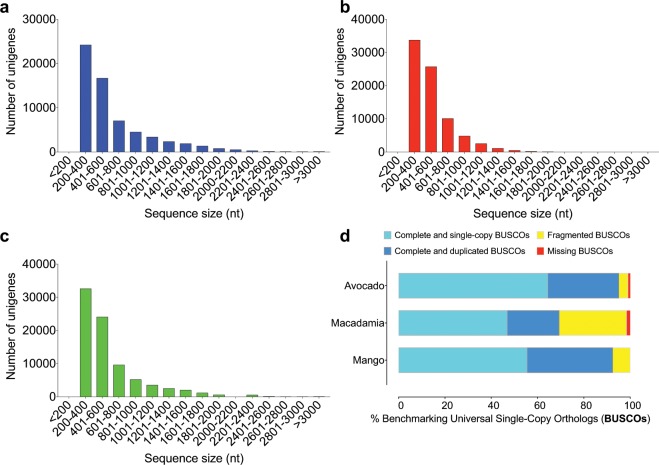
Sequence length distributions and assessment of completeness of the avocado, macadamia and mango unigenes. (**a**–**c**) Sequence length distributions, (**d**) transcriptome completeness as determined by Benchmarking Universal Single-Copy Orthologous (BUSCO). The figure was generated using GraphPad Prism Version 7.0a.

Read quality assessment and by extension, read validation was done using FastQC, quality control (QC) plots were aggregated using MultiQC^22^ and are presented in Fig. 2. We used HISAT2^35^ to map avocado and macadamia RNA-Seq reads to their respective reference genome assemblies^10,11^. 73,7 and 79,8% of the avocado and macadamia reads mapped to their respective reference genome assemblies (Table 1). 63420, 78871 and 82198 unigenes of avocado, macadamia and mango were generated from the RNA-Seq data using a combination of *de novo* transcriptome assembly and redundancy reduction (Fig. 1; Table 2). We used BLASTn (e-value cut-off of 1e-5 and an identity cut-off of 70%) to compare our avocado and macadamia unigenes to the published reference gene sets^10,11^. 22670 (92%) and 27322 (77%) of the reference avocado and macadamia genes respectively were present in our assemblies (Table 1). The length distribution of “unigenes” was similar across the three species (Fig. 3a–c).

**Table 2:** *De novo* assembly statistics of avocado, macadamia and mango transcriptomes before (Trinity output) and after redundancy reduction (Unigenes).

	Avocado		Macadamia		Mango	
	Trinity output	Unigenes	Trinity output	Unigenes	Trinity output	Unigenes
# contigs (>=0 bp)	249765	63420	225591	78871	251204	82198
# contigs (>=1000 bp)	42988	10981	17643	4464	44854	10694
# contigs (>=5000 bp)	28	2	0	0	14	1
Total length (>=0 bp)	154556593	41442153	106195638	40705830	156057297	49246959
Total length (>=1000 bp)	69201144	16247577	23529519	5499159	72163715	15228411
Total length (>=5000 bp)	153870	11058	0	0	76292	5547
# contigs	100110	28816	68025	29090	98975	34564
Largest contig	6121	5700	3594	3219	6179	5547
Total length	109464144	28572369	58255825	22035423	110183488	31425165
GC (%)	43.33	46.89	45.09	48.29	41.82	45.58
N50	1239	1104	888	756	1292	978
N75	817	744	663	606	839	675
L50	29949	9111	23589	10869	29822	11184
L75	57262	16985	42633	19050	56299	20938
# N’s per 100 kbp	0	0	0	0	0	0

Transcriptome assembly validation was done using Benchmarking Universal Single-Copy Orthologs (BUSCO) v. 3^36^. 70–95% of complete BUSCOs were present in the three *de novo* transcriptomes indicated high-quality assemblies, particularly for avocado and mango transcriptomes (Fig. 3d). Our normalized avocado assembly lacks only 3 while our normalised mango assembly has all near-universal single-copy genes (Fig. 3d). BUSCO provides a quantitative measure of transcriptome quality and completeness, based on evolutionarily-informed expectations of gene content from the near-universal, ultra-conserved eukaryotic proteins (eukaryota_odb9) database^36–38^. The BLASTx program (e-value cut-off of 1e-3) was used to annotate the “unigenes” based on UniProtKB/Swiss-Prot, a manually annotated, non-redundant protein sequence database^26,27,39^. 64–67% of the “unigenes” per species were annotated to the UniProtKB/Swiss-Prot non-redundant protein sequence database. A comprehensive workflow and links to obtain transcriptome data are provided. This dataset adds valuable transcriptome resources for further study of developmental gene expression, transcriptional regulation and functional genomics in avocado, macadamia and mango.

## Supplementary information

Figure S1

## Data Availability

**Trimmomatic v. 0.35 parameters:** *trimmomatic-0.35.jar PE -phred33 in_forward.fq.gz in_reverse.fq.gz out_forward_paired.fq.gz out_forward_unpaired.fq.gz out_reverse_paired.fq.gz out_reverse_unpaired.fq.gz ILLUMINACLIP: TruSeq3-PE.fa:2:30:10 LEADING:3 TRAILING:3 SLIDINGWINDOW:4:15 MINLEN:36* **HISAT2 v 2.1.0 parameters:** *hisat2-build reference_index_name genome.fa* *hisat2 –x reference_index -1 reads_1a.fq,reads_1b.fq, reads_1c.fq,reads_1d.fq -2 reads_2a.fq,reads_2b.fq,reads_2c.fq,reads_2d.fq -S output.sam* **SamTools v. 1.9.0 parameters:** *samtools view -b -o output.bam samfile_from_hisat2.sam* *samtools sort -o sorted.bam output.bam* *samtools depth sorted.bam* | *awk ‘{sum*+=$*3} END* {*print “Average* = *”,sum*/*NR*}’ **Trinity v. 2.7.0 parameters:** *Trinity* --*seqType fq* --*left reads_1a.fq,reads_1b.fq,reads_1c.fq,reads_1d.fq* --*right reads_2a.fq,reads_2b.fq,reads_2c.fq,reads_2d.fq* --*CPU 6* --*max_memory 20G* **CD-HIT-EST v. 4.8.1 parameters:** *cd-hit-est –i trinity_transcripts.fasta –o output file –c 0.9*. **TransDecoder v.5.5.0 parameters:** *TransDecoder.LongOrfs -t cd-hit-est__0.95_transcripts.fasta* **BUSCO v. 3 parameters:** *python BUSCO.py -i unigenes -l OrthoDB v9 -o output_name* **BLAST v. 2.7.1 parameters:** *makeblastdb -in reference_trancriptome assembly.fasta -dbtype “nucl”* *blastn -query unigenes.fasta -db reference_trancriptome assembly.fasta -out outputfile.txt -evalue 1e-5 -max_target_seqs. 20 -outfmt 6* *makeblastdb -in -in uniprot_sprot.fasta -dbtype “prot”* *blastx -query unigenes.fasta -db uniprot_sprot.fasta -out outputfile.txt -evalue 1e-3 -max_target_seqs. 20 -outfmt 6*.
