# Supplementary material for: De novo transcriptome assembly and annotation for gene discovery in avocado, macadamia and mango
Source: Sci Data. 2020 Jan 8;7:9. doi: 10.1038/s41597-019-0350-9 (PMC6949230; doi:10.1038/s41597-019-0350-9)
Supplement: Supplementary file 1 — Figure S1 [file 41597_2019_350_MOESM1_ESM.pdf]

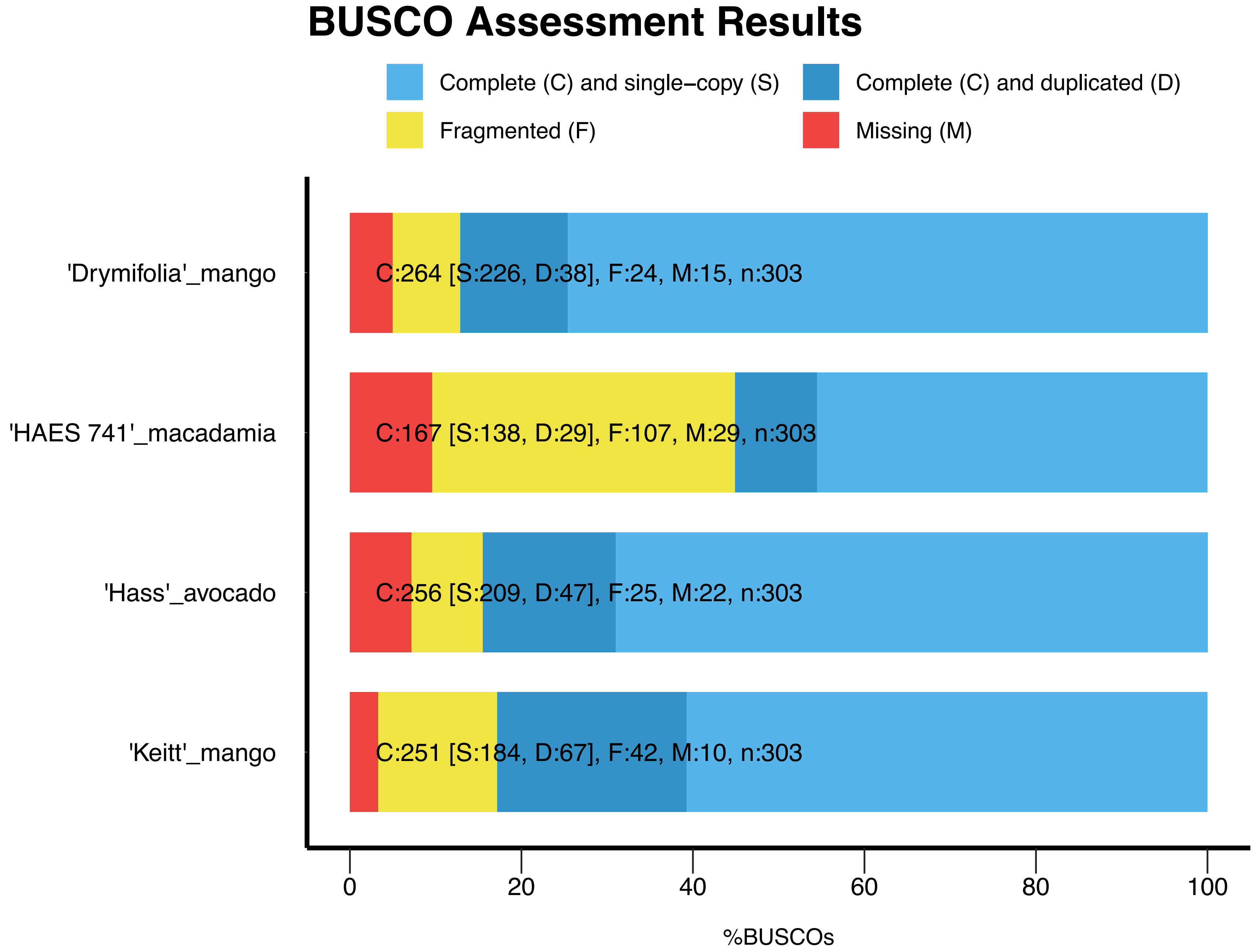

Figure S1. Sequence Assessment of the completeness of the reference 'Hass' and 'Drymifolia' avocado [10] and 'HAES 741' macadamia [11] protein-coding genes and 'Keitt' mango transcriptome assembly [9] by BUSCO.
